# Increasing Mu wave desynchronization after dance classes on people with Parkinson’s disease

**DOI:** 10.3389/fnhum.2025.1443730

**Published:** 2025-03-24

**Authors:** Jade Thalia Rodrigues Vilhalva, Breno Cesar de Oliveira Imbiriba, Lane Viana Krejcova, Carlomagno Pacheco. Bahia

**Affiliations:** ^1^Laboratório de Neuroplasticidade, Instituto de Ciências da Saúde, Universidade Federal do Pará (UFPA), Belém, Pará, Brazil; ^2^Faculdade de Meteorologia, Instituto de Geociências, UFPA, Belém, Pará, Brazil; ^3^Programa Interdisciplinar Trópico em Movimento, Reitoria, UFPA, Belém, Pará, Brazil; ^4^Faculdade de Dança, Instituto de Ciências da Arte, UFPA, Belém, Pará, Brazil

**Keywords:** Mu-rhythm, event-related desynchronization, Parkinson’s disease, mirror neuron system, dance

## Abstract

This pilot study attempted to identify a relationship between dance and mirror neuron activity in people with Parkinson’s disease by investigating Mu rhythm desynchronization in electroencephalographic (EEG) data before and after regular participation in a program of dance classes. During the EEG recordings, the participants observed a sequence of videos showing either choreographic (complex) or daily (simple) movements, each preceded by a baseline image (dark screen) and a control video (moving blocks). The results showed a statistically significant increase in Mu rhythm desynchronization in the alpha 1 band at the central channels after 6 months of dance classes. Control comparisons with occipital channels showed no such increase. Mu rhythm suppression has been demonstrated to reflect the activity of the human mirror neuron system, respond to variations in motor expertise, and seem to be impaired in Parkinson’s disease. The Mu wave desynchronization increase shown here, after 6 months of dance classes, is an objective measurement of the benefits of such practice for people with Parkinson’s disease (PD).

## 1 Introduction

Parkinson’s disease (PD) is a progressive neurodegenerative disorder that affects brain regions where neural circuitry is responsible for controlling voluntary movements and is involved in cognitive processing and emotional behaviors (Marsden, 1982; Mink, 1996; O’Reilly and Frank, 2006). PD presents a variety of motor and non-motor symptoms, all associated with basal ganglia circuitry dysfunction caused by the loss of dopamine-producing cells in the substantia nigra pars compacta. Several studies have attempted to explain the relationship between the basal ganglia and mirror neuron activity, particularly in PD (e.g., Alegre et al., 2011). Mirror neurons (MNs) are a set of neurons first observed by Rizzolatti et al. (2001) using single-cell recording. They discovered a set of neurons in the macaque premotor cortex (Area F5) that appeared to respond both when a monkey performed a given action and when the animal remained motionless, observing another individual performing a similar action (Di Pellegrino et al., 1992). Numerous studies involving neuroimaging and electroencephalography (EEG) have shown robust evidence for a human homolog to the monkey mirror system, making MNs a subject of great interest in the field of social neuroscience (Rizzolatti and Sinigaglia, 2016). Now, we know that MNs can be activated in humans when a person performs or observes a given action, thus performing an “internal” simulation of the observed acts. This is a fundamental process in understanding how the brain is able to encode others’ actions and intentions, affirming its relationship with more complex hierarchical circuits of cognition—such as understanding human intent, the so-called Theory of Mind (e.g., Siegal and Varley, 2002).

The activity of mirror neurons is influenced by previous motor experience and can be easily assessed through electroencephalography (EEG) recordings by detecting changes in the Mu band wave amplitudes (Mu rhythm) during action observation (Altschuler et al., 1997; Heyes and Catmur, 2022). Mu rhythm desynchronization occurs in the usual alpha wave band (8–13 Hz) but differs from alpha waves due to its amplitude variability during the observation of motor acts and localization (Pfurtscheller et al., 1997), primarily at the central and parietal channels (Pineda, 2005; Fox et al., 2016). It is evident that Mu rhythm desynchronization and MN activity are associated and related to sensorial, motor, and cognitive functions (Muthukumaraswamy and Johnson, 2004; Gallese, 2008; Martineau et al., 2008).

Although there have been recent critiques regarding the use of the Mu rhythm as an index for neural mirroring, careful analysis performed by Bowman et al. (2017), along with thoughtful reviews and meta-analyses (Hobson and Bishop, 2016; Fox et al., 2016), has shown that, despite the need for caution when interpreting claims about mirror system activity in experimental setups where Mu rhythm desynchronization can be confounded with alpha activity, when properly observed in experimental designs that (a) include EEG activity from multiple electrode sites, (b) evaluate potential domain-general visual and attentional confounds, and (c) establish a basic mirroring property in the action observation conditions, Mu rhythm, by its property of suppression during action execution and observation, can be used as a reliable index to assess MN activity. This pattern of desynchronization seems to change in some neuropsychiatric or even neuropathological conditions, such as Parkinson’s disease (Defebvre et al., 1994), which is the focus of this study.

Evidence demonstrates that changes in Mu wave desynchronization—suggesting MN activation—occur when observing the execution of previously learned movements. This desynchronization happens with visual stimulation related to known movements. It appears that only actions mastered by the observer can be mirrored. For example, Heida et al. (2014) showed that event-related Mu rhythm desynchronization during movement observation, as measured with EEG, was altered in PD, and these changes may be related to symptoms such as impaired motor learning and programming. Alegre et al. (2010) showed in a study using deep brain stimulation in individuals with PD that movement observation generated a decrease in both the abnormal beta activity observed in the subthalamic nucleus (STN) in participants with PD and in the cortico-STN coherence, indicating that the STN is influenced by the activity of the human mirror system. However, such input by itself is probably insufficient to generate feedback output to the cortex.

Physical exercise enables adaptation in certain areas of the central nervous system, such as the basal ganglia (Morgan et al., 2015). In PD patients, physical exercise affects both motor and non-motor symptoms (Speelman et al., 2011), although no clear exercise type seems to be most beneficial (Amara and Memon, 2018). In recent years, several studies on the qualitative effects of dance practice on people with PD have been performed, (e.g., Westheimer, 2008; Rocha et al., 2017; Shanahan et al., 2017). An electroencephalographic assessment of the effects of dance practice on people with PD has shown a global increase in both alpha peak power and frequency after just one dance class (Levkov, 2015).

The present study aimed to observe changes in Mu rhythm ERD via EEG recordings during the observation of two types of movements in people with PD, before and after participation in regular, 6-month-long, twice-weekly dance classes. The hypothesis of this work is that, after dance classes, Mu rhythm desynchronization will increase, providing an objective EEG-based assessment of the effects of dance on PD symptoms.

## 2 Methodology

### 2.1 Participants and dance intervention

This was an uncontrolled pilot study. Initially, a total of 30 participants were enrolled (January–March 2018) in dance sessions held twice a week by the “Parkinson Group” and staff from UFPA’s Neuroplasticity Laboratory (Instituto de Ciências da Saúde, Av. Generalíssimo Deodoro 1, Belém, PA, Brazil).

The participants were recruited through social media announcements and spontaneous demand. For enrollment in the study, the inclusion criteria included patients diagnosed with idiopathic PD according to the criteria of the UK Parkinson’s Disease Society Brain Bank, have been receiving pharmacological treatment for at least 3 years, who were at Hoehn and Yahr stage I to III, and who were physically fit to participate in dance classes, as attested by a physician. Participants unable to engage in dance activities, patients with osteoporosis or any condition that could pose a risk for physical activity, and patients with other neurological or neuropsychiatric conditions were excluded.

**Figure 1:**
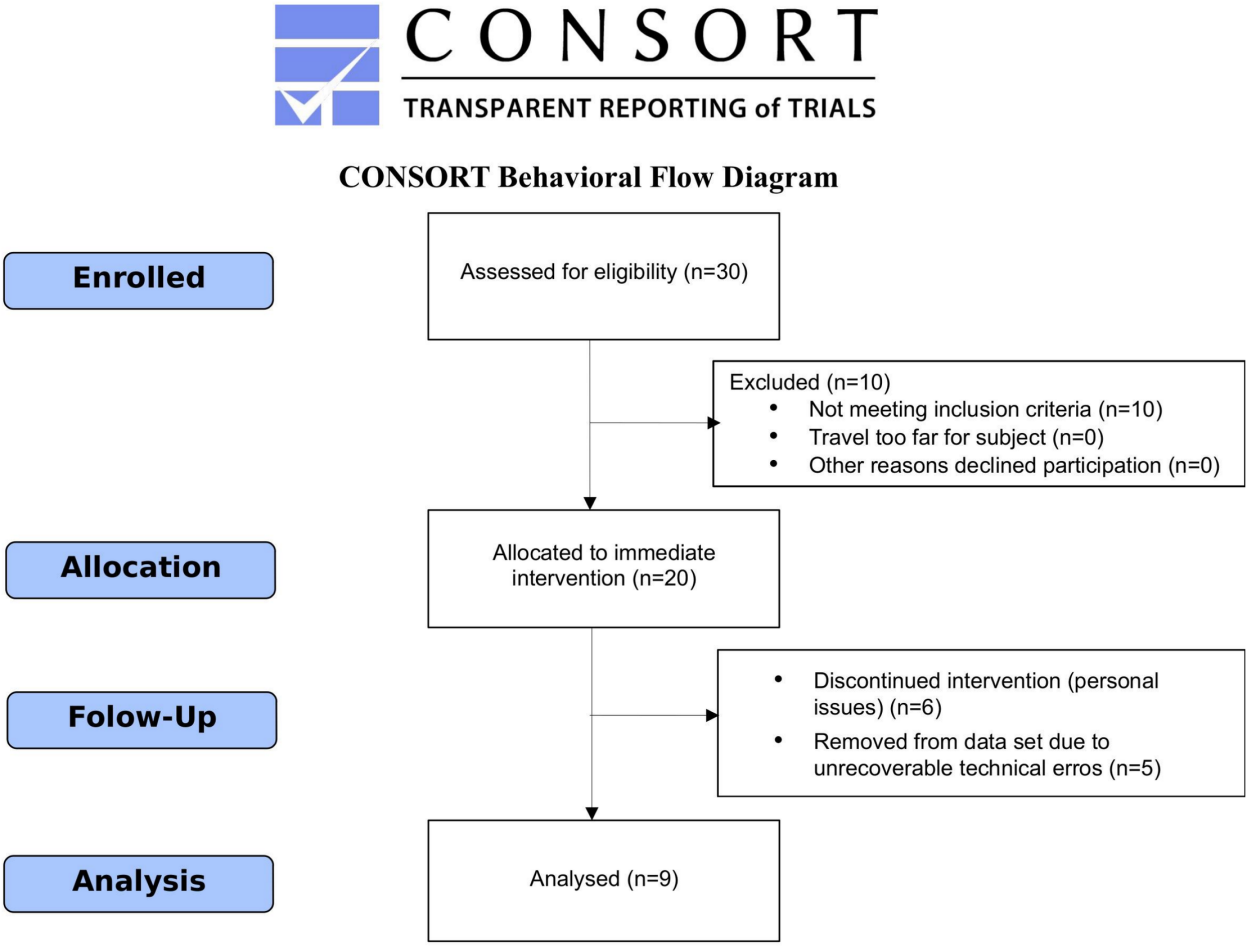
CONSORT flow diagram of the progress throughout the phases of the study, showing the total number of enrolled, excluded, and analyzed individuals.

For the screening process, all participants completed a form on our website and were contacted to schedule a preliminary evaluation with our investigators. They were instructed to bring all medical documentation they possessed regarding their condition, such as certificates, reports, prescriptions, and previous exams. All participants were evaluated by a neurologist to confirm the inclusion criteria and undergo the MDS-UPDRS evaluation and by a physician to confirm their eligibility to participate in the dance classes. The consent form was presented to the participants by the physician, and any questions were addressed before the participant or caregiver signed it. All participants agreed to take the dance classes for at least 10 months. Of the participants, 10 were excluded for having mobility issues. Two EEG recordings were performed for each participant: the first one just before the motor activity sessions began in March 2018 and the second one after 8 months of regular bi-weekly participation in the motor activity sessions, in November 2018. During this process, six participants decided to withdraw from the intervention for personal reasons, and five participants were excluded due to irretrievable technical issues with the data. Ultimately, a total of nine participants, including five female participants, aged 62.9 ± 7.1 years, with varied clinical scenarios (UPDRS mean of 57.9 ± 18.3 points) and functional status ratings ranging from 2 to 4 on the Hoehn and Yahr scale, participated in this study. See Figure 1 for CONSORT flow diagram.

All participants attended dance classes using the Baila Parkinson method (Krejcova et al., 2017; Duarte et al., 2023). This method incorporates various dance styles with movements adapted for people with Parkinson’s disease, organized into five work lines focused on different aspects of PD symptomatology: Motor, cognitive, neuropsychiatric, somatosensory, and socialization. The method combines elements from different dance styles such as ball dance, classic ballet, folk dances, and samba. The session follows an artistic-themed script, incorporating the constant use of mental imagery and rhythmic choreographic movements.

The dance sessions lasted between 40 and 50 min and were based on an artistic repertoire, structured as a themed dance class. Each session’s theme (e.g., *The Blue Danube; Romeo and Juliet; The Phantom of The Opera*) was divided into at least five acts to ensure that all the work lines of the method were addressed. Then, the acts were staged by the participants as a collective theatrical dance performance, starting with a choreographed warm-up that introduced the first act of the story. The subsequent acts followed with increasing intensity, with the fourth act typically focusing on the motor work line and the fifth act concluding the story with a cool-down activity. Throughout the session, mental imagery was a constant element in the development of the dance class, with each participant assuming the role of the protagonist of the act. The dance style applied varied according to the theme of the class. An example of a dance session planning script and the activities performed can be found in the Supplementary material.

This study was approved by the research ethics committee of the Hospital Universitário João de Barros Barreto (CEP-HUJBB) – Universidade Federal do Pará (Protocol n° 49347115.0.0000.0017).

### 2.2 EEG data acquisition description

Electroencephalography was used to detect the rhythm modulation of cortical activity induced by the observation of motor actions from different modalities, using a modified version of the protocol described by Orgs et al. (2008). All EEG recordings followed the international 10/20 system, using the Cz electrode as the reference and a metopic electrode as the ground. The EEG recording device used was an EMSA BNT 36, with an electrode impedance of 5 *k*Ω and an acquisition rate of 200 Hz.

### 2.3 EEG protocol

**Figure 2:**
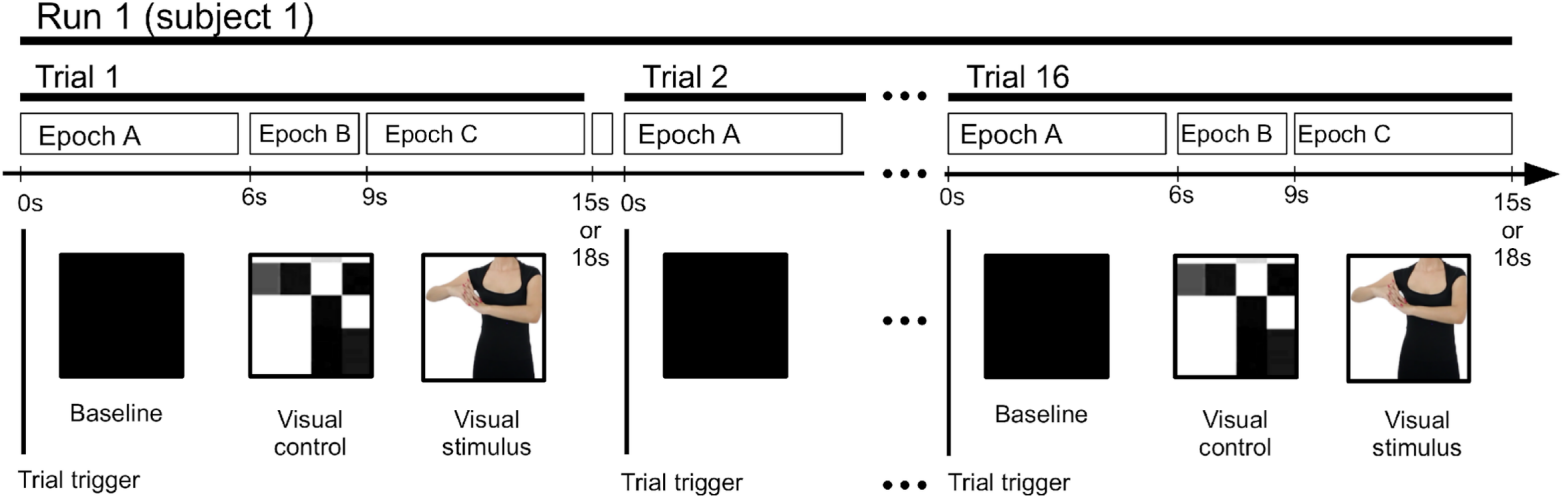
Time layout of the epochs and trials in each EEG run.

Each participant’s EEG recording (an EEG “run”) was composed of a continuous sequence of 24 trials, each recorded during the presentation of a video stimulus lasting from 15 to 18 s and separated by an independent trigger channel. The trials’ structure is shown in Figure 2. The trials were made up of three consecutive sections (epochs).

During Epoch A, the visual baseline, a 6-s long black screen was presented with no additional visual stimulus. During Epoch B, the visual control, a 3-s long display of moving blocks was presented, created from an extremely low-resolution version of the actual (see Epoch 3 below) stimulus video. It maintained the same movement patterns, lighting, contrast, and other visual conditions of the following stimulus, except for the absence of biologically co-specific movements (Orgs et al., 2008).

During Epoch C, the visual stimulus, a 6- to 9-s long video was presented, displaying an actor performing one of two types of movements: typical movements of daily activities (“daily”) and choreographic dance-type movements (“chor”), both performed by dance professionals. The video showed only the actor’s neck to waste area, focusing on the arms, hands, and chest, while excluding other body parts to reduce distractions, ocular exploratory movements, and the activation of face recognition-related cognitive processes. Actual movements shown in a particular trial were randomly selected. All choreographic dance-type movements presented to the participants were the same movements used during the dance classes. An unspecified break was taken between each trial.

Here, all stimuli were visual in nature. Hence, the rationale behind the selection of these three epochs was to be able to separate generic visual stimuli from motor visual stimuli. Epoch A had no visual stimuli and served as a baseline, Epoch B had non-cognitive visual stimuli, which should be observable at the occipital channels but not at the central channels, and Epoch C had clear motor visual stimuli, which should also be observable at the central channels.

### 2.4 Data filtering

A range of electrical noise artifacts were noted in the data. Noises in EEG recordings usually have common sources, such as instabilities of electrode impedance, issues with cables or the recording device, or ground connection problems. To remove such noises, filters, data trimming, wavelets, and ICA methods were used and are detailed in the Supplementary material. Below is a sketch of the method used.

Initially, a simple 1.5 Hz high-pass filter and a 60 Hz notch filter were applied to the data to remove the electrical grid generation frequency and slow power fluctuations. Voltage readings greater than 3 volts were trimmed with a 1 s window. Alpha band selection was performed using a fourth-order Symlet 9 discrete wavelet transform (DWT), keeping only signals within the nominal range of 6.5 Hz to 25 Hz. As electrical noise introduced extra power to the signal, a power variability comparison was performed across all trials of each run to identify and remove trials beyond 3σ from the mean.

Independent component analysis of the data was performed on each EEG data series, and despite the DWT filtering, eye blink bioelectrical noise could still be observed in the ICA activations. A spatial localization test was conducted to detect eye blink-related activations associated with the Fp1 and Fp2 frontal channels. Furthermore, activations showing high-frequency noise or power contamination were also filtered out.

### 2.5 Statistical analysis

After the data filtering and trimming processes, each EEG time series was decomposed into trials and then into three epochs, producing a total of 61 trials for the test phase and 67 trials for the retest phase. Epochs A and B had durations of 6 and 3 s, respectively. Epoch C varied from 6 to 9 s, but for uniformity, only the first 6 s were taken into account. To estimate the EEG wave power, two spectral bands were selected: from 7.5 Hz to 10.5 Hz (alpha 1 waves, α_1_) and from 10.5 Hz to 13 Hz (alpha 2 waves, α_2_) (Cochin et al., 1999). Each epoch was then filtered into these two bands using a boxcar window in the frequency domain, and its total power (i.e., the sum of the squared amplitudes) was computed. To compare the power of the epochs from different EEG data sets (different runs), the natural logarithm of the power was used. In each trial, three quantities were computed: the difference in the logarithm of the power between epochs A and B, between epochs A and C, and between epochs B and C (Equations 1–3). This is similar to the definition of “task-related power” (TRP), and this term is retained here. TRP was then computed for each spectral band, *w* (α_1_ and α_2_).


(1) $$TRP_{w}^{\mathrm{AB}}=\log_{10}\left(E_{w}^{A}\right)-\log_{10}\left(E_{w}^{B}\right)$$

(2) $$TRP_{w}^{\mathrm{AC}}=\log_{10}\left(E_{w}^{A}\right)-\log_{10}\left(E_{w}^{C}\right)$$

(3) $$TRP_{w}^{BC}=\log_{10}\left(E_{w}^{B}\right)-\log_{10}\left(E_{w}^{C}\right)$$

The interpretation of TRP is simple: a reduction in power between two epochs is represented by a negative TRP value, representing desynchronization.

The computed TRP values for a particular transition (AB, BC, or AC), band (alpha 1 or 2), and phase (test or retest) were grouped into a set containing the results of all available trials. For each of these sets, a central value and an error estimate were computed. The central value was the average of the data contained in a 2-sigma-wide window around the median value (mean around the median). The error estimate was calculated by dividing the standard deviation by the square root of the number of observations. Statistically significant differences in these TRP sets were computed using Student’s *t*-test.

### 2.6 Channel comparison

Desynchronization is characterized by a reduction in power from one task to another; therefore, a reduction in TRP is expected at the occipital channels when the video changes from a blank screen to a moving block screen. Similarly, desynchronization is expected in the central channels when a recognizable movement appears on the screen, transitioning from the moving block screen to the high-definition movement videos. In this study, the occipital channels used were O1, Oz, and O2, and the central channels used were C1, Cz, and C3. Each group was then averaged to form a single number, which was then processed (mean around the median) across all trials.

## 3 Results

### 3.1 Mu rhythm desynchronization

The desynchronization levels for the alpha 1 (*α*_1_) waves across the AB and BC transitions, before and after the dance classes, for both types of movements (dance movements and daily movements) are shown in Table 1. As expected, the AB transition from the black screen to the video of moving blocks showed no statistically significant change in desynchronization, whereas for the BC transition, a significant change in desynchronization was observed, with *p*-values less than 0.05 for both types of movements. These results were consistent with mirror neuron activation during the observation of motor movements.

**Table 1:** Central channels: statistical differences in the TRP values between the test and retest of the participants after motor training in dance.

Channel	Type	Wave	Case	*p*-value	Rel.	Test	Error	Retest	Error
C	dance	α1	AB	0.248		−0.068	0.023	−0.134	0.0323
C	daily	α1	AB	0.358		−0.100	0.022	−0.072	0.0210
C	dance	α1	BC	0.014	**	−0.039	0.016	−0.091	0.0179
C	daily	α1	BC	0.010	**	−0.027	0.019	−0.099	0.0200
C	dance	α2	AB	0.92		−0.139	0.022	−0.157	0.028
C	daily	α2	AB	0.049	*	−0.0873	0.025	−0.151	0.020
C	dance	α2	BC	0.388		0.0210	0.017	0.004	0.022
C	daily	α2	BC	0.334		−0.0251	0.017	0.000	0.020

Mean and standard deviation values are shown for both the test and retest (last 4 columns). A significant difference between the test and retest was found only in the B/C transition. *p ≤ 0.05, **p ≤ 0.01.

An increase in alpha 1 wave desynchronization was observed for both choreographic and daily-type movements at the central channels during the (stimulus) BC transition (*p* < 0.014) in the central electrodes, with a significant difference noted in the BC transition between the periods before and after the intervention (Table 1).

**Figure 3:**
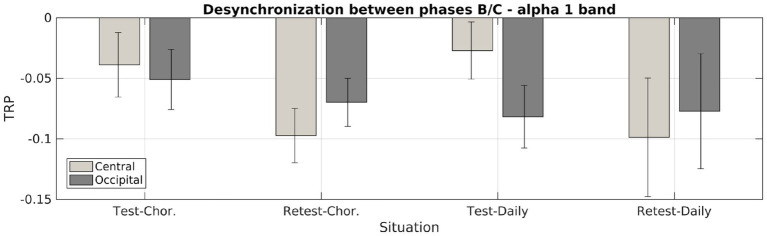
Comparison of alpha 1 wave desynchronization in the central and occipital channels, before and after the dance therapy. TRP averages indicate an increase in desynchronization in the central channels after months of motor training in dance, without a significant increase in desynchronization of the occipital channels, indicating a possible positive effect on desynchronization regarding cognitive capacity.

A similar analysis was performed using the data from the occipital channels as input to test for similar effects on those channels, as shown in Table 2. No transition exhibited statistically significant desynchronization, contributing to the interpretation of Mu rhythm desynchronization. The effects of alpha 1 wave desynchronization for both central and occipital channels are represented in Figure 3, where bars and error bars indicate the mean around the median and the normal mean estimation error, respectively. Increases in alpha 2 wave desynchronization were not apparent in the analyzed data, as shown in Tables 1, 2.

**Table 2:** Occipital channels: statistical differences in the TRP values between the tests and retests of the participants after dance training.

Channel	Type	Wave	Case	*p*-value	Rel.	Test	Error	Retest	Error
O	dance	α1	AB	0.305		−0.116	0.022	−0.169	0.028
O	daily	α1	AB	0.526		−0.114	0.027	−0.137	0.026
O	dance	α1	BC	0.439		−0.044	0.019	−0.065	0.017
O	daily	α1	BC	0.879		−0.075	0.021	−0.077	0.011
O	dance	α2	AB	0.893		−0.166	0.020	−0.179	0.030
O	daily	α2	AB	0.104		−0.141	0.025	−0.194	0.020
O	dance	α2	BC	0.540		0.0093	0.019	0.0194	0.019
O	daily	α2	BC	0.003	**	−0.038	0.018	0.0317	0.022

No significant difference was observed between the test and retest. **p ≤ 0.01.

## 4 Discussion

In this study, we compared Mu rhythm desynchronization in individuals with PD who attended dance sessions, under two different types of visual stimuli: choreographic dance-type movements and typical movements of daily activities. Our initial hypothesis predicted that the amount of Mu rhythm suppression would be significantly affected by dance activities in PD patients between the periods before and after the intervention, with a remarkably increased effect for choreographic (complex) movements. This is in accordance with the assumption that Mu suppression is induced by MNs during action observation (see Pineda, 2005 for a review).

Our results showed that the effects of such motor activities on the increase in alpha 1 wave desynchronization were observed for both choreographic and daily-type movements at the central channels during the (stimulus) BC transitions (*p* < 0.014), corresponding to central brain areas.

This is in line with the findings of previous studies on motor training-induced Mu-ERD changes (Park et al., 2018) and the effects of movement on higher Mu-rhythm desynchronization (Wagner et al., 2012).

Our results also showed no clear distinction between the two types of visual stimuli on the change in TRP. Contrary to our hypothesis, Mu-ERD seemed to be similarly affected when observing both daily (simple) and choreographic (complex) movements. No increase was observed in the (control) AB transitions and in any of the occipital channels, indicating that dance activities might have a direct effect on Mu rhythm desynchronization for both choreographic and daily movement categories.

Despite our previous expectations, the effects of dance classes on both daily and choreographic movements may have been linked to the characteristics of the intervention, albeit in a non-specific manner. It is widely known that dancing induces high activation of the MN system as action imitation comprises the main activity of a dance class (Zardi et al., 2021) and that MN activity is influenced by an increase in the individual’s motor repertoire (Orgs et al., 2008; Calvo-Merino et al., 2005; Cannon et al., 2014). In addition, increasing motor skills should be expected to be transferable to daily activities through easy adoption of motor strategies. Therefore, it should be expected that the inclusion of choreographic movements in a person’s skill set would also induce an improvement in their daily movement skills.

Therefore, the augmentation of the motor repertoire through dance classes could impact Mu rhythm desynchronization for all kinds of visual motor stimuli. This finding is in line with results from previous studies that reported modulation of activity in motor areas and mirror neuron activation/Mu-ERD induced by motor experiences. Data noise and the small population sample likely contributed to the negative result for α2 wave desynchronization.

Moreover, when considering the specific characteristics of the Baila Parkinson intervention for the observed results, one of the strongest characteristics of the method is the constant use of imagery as a strategy to improve movement. Trained dancers are experts in movement imagery, and both motor imagery and visual imagery are frequently used as tools in dance training to improve movement quality, induce artistic expression, memorize choreographic sequences, and create new movements (See, for example, Pavlik and Nordin-Bates, 2016). Motor imagery is also a strong element in the induction of MN activation (Abbruzzese et al., 2015; Caligiore et al., 2017). Taken together, these elements could have been responsible for the effects on Mu-ERD observed in the results from the periods before and after the intervention in this study.

Our results may have important implications for the rehabilitation and treatment of PD. It has been shown that people with PD show impaired Mu rhythm desynchronization during movement observation and that this impairment may be a marker for PD (Heida et al., 2014). In people with PD, Mu-ERD during action observation is impaired even at early stages (Heida et al., 2014) and corresponds to decreased cortical cerebral electric activity, which seems to be recovered by administering dopamine therapy and/or by the activation of other cortical cerebral areas through compensatory mechanisms (Dujardin et al., 1999). Direct recordings through DBS electrodes in people with PD demonstrate selective ERD in beta waves within the subthalamic nucleus (STN) during action observation. This suggests, together with the well-known connections between the STN and cortical mirror neuron areas, that STN oscillatory activity is involved in mirror neuron activity in humans and is therefore modulated by training (Alegre et al., 2010; Marceglia et al., 2009).

Although the present study did not assess clinical improvement in participating individuals, a parallel study (Duarte et al., 2023) performed on the very same group of patients during the same intervention and time period revealed significant improvements in movement (balance and gait) and executive functions, especially in cognitive flexibility and inhibitory control. This indicates that the Mu rhythm changes observed here, which suggested MN system improvement, occurred alongside improvements in actual movements, thereby strengthening the assumed link between the Mu rhythm, MNs, and movement.

Taken together, these findings suggest that the dance classes provided to the participants led to a statistically significant improvement in Mu-rhythm cortical activity usually associated with the mirror neuron system, which could be directly linked to the clinical improvements observed in people with PD following the dance classes.

In summary, our results indicate that dance activities modulate Mu rhythm-ERD during the observation of both daily (simple) and choreographic (complex) movements. Therefore, the augmentation of the motor repertoire through dance classes could impact Mu rhythm desynchronization for all kinds of visual motor stimuli. This finding is in line with results from previous studies that reported the modulation of activity in motor areas and mirror neuron activation/Mu-ERD induced by motor experiences. Data noise and the small sample size likely contributed to the negative result in *α*_2_ wave desynchronization.

## 5 Limitations

This was a pilot study that was conducted in a single center with a small number of participants. Although our results confirmed previously described events regarding Mu activity and motor repertoire, specific features such as data noise and individual baselines might have affected the data. Nevertheless, the study obtained consistent statistical results. Other limitations include possible confounding effects of certain PD subtypes, disease severity, and the effects of levodopa treatment, dosage, and time since drug administration when performing the tests. Furthermore, although this study targeted patients diagnosed with PD using the Brain Bank criteria, the confirmation of PD diagnosis could only occur postmortem. Therefore, it cannot be ruled out that some patients with a mistaken diagnosis were included. Lastly, in contrast to cross-sectional studies, longitudinal studies are needed in future research, including those performed on a large scale and across all stages of PD.

## 6 Conclusion

The results presented above suggest that dance classes could be potentially effective at influencing mirror neuron system activity, contributing to changes at the alpha wave desynchronization levels (Mu rhythm). The dance classes provided to the participants had an overall beneficial effect on their quality of life and movement skills. Mu rhythm suppression is an easily accessible measure of MNS activity, which can act as a tool for neurorehabilitation itself or for assessing the effects of therapeutic approaches in PD.

## Data Availability

The raw data supporting the conclusions of this article will be made available by the authors, without undue reservation.
