# Supplementary material for: Increasing Mu wave desynchronization after dance classes on people with Parkinson’s disease
Source: Front Hum Neurosci. 2025 Mar 24;19:1443730. doi: 10.3389/fnhum.2025.1443730 (PMC11973361; doi:10.3389/fnhum.2025.1443730)
Supplement: Supplementary file 1 [file Data_Sheet_1.pdf]

Supplementary Material

1 Data Filtering

1.1 EEG Filtering

A range of electrical noise artifacts were observed in the data. Noises in EEG recordings usually have common sources, such as instabilities of impedance of electrodes, cables recording device or ground connection. To remove such noise, filters, data trimming, wavelets and ICA methods were used. Initially, a 1.5Hz high-pass filter and a 60Hz notch filter were applied to the data in order to remove the electrical grid generation frequency and slow potential fluctuations. Following, any feature with amplitude higher than 3 volts was removed from the data by trimming the whole EEG recording 500ms before and after the feature.

1.2 Wavelets

Wavelet techniques have been used for EEG denoising and feature detection, for example see (Herrmann, Grigutsch & Busch 2005) for an introduction, however the type of wavelet to be used depends on applications and there is no consensus on best practices. For epileptic feature detection, a review on previous studies (Faust et al. 2015) demonstrate the DB4 discrete wavelet transform (DWT) as being the most used, but it is far from being the only one. For example, (Malaver 2017) determines that best detection of epileptic features occurred using 6-level Bior3.9 DWT. Other situations ask for different wavelets. In (Scolaro et al. 2013), epileptic features are best detected with RBio2.8 and Coif4, but to filter EEG background noise Bior3.1 seems best. For denoising of healthy subjects' EEGs, (Mamun, Al-Kadi & Marufuzzaman 2013) finds DB8 as the best DWT option. Also, new types of wavelets may be created specially to fit a particular purpose (Glassman 2005). With respect to the number of levels, (Gandhi, Panigrahi & Anand 2011) suggests that for DWT, from level 4 and above there is a 90% accuracy in feature classification, and (Subasi 2007) uses 5-level DWT to classify epileptic events. For general EEG filtering (Al-Qazzaz et al. 2015) surveyed 26 types of wavelets applied to denoise EEG representing "working memory", and the SURE thresholding technique, to finding that Symlet 9 is most correlated with original data. In this work we are not interested in particular features but in filtering large sections of EEG data, hence Symlet 9 DWT was used.

1.3 DWT Filtering

Filtering was performed on the whole time series for each EEG channel using a fourth-order Symlet 9 Discrete Wavelet Transform (DWT), yielding five "nominal" frequency bands, each one with a different type of energy source, either biological or external. Bands, frequencies and Sources are shown on Table 1.

1.4 Trial elimination

After basic DWT filtering a power variability analysis was performed on all trials of each EEG run. For the ith trial in a run, the mean absolute deviation (MAD) of the power, $\sigma(P_i)$, was computed: $\sigma(P_i) < \langle x_i - \langle x_i \rangle \rangle$. Collecting all trials from the same EEG run, a threshold value was defined as the median of all power deviations, $\text{med}(\sigma(P_i))$, in that run plus three times the MAD of

the power deviations themselves, $\sigma\left(\sigma\left(P_i\right)\right)$. Trials were kept only if their deviation $\sigma\left(P_i\right)$ did not exceed this threshold, i.e., $\sigma\left(P_i\right) < \text{med}\left(\sigma\left(P_i\right)\right) + 3\sigma\left(\sigma\left(P_i\right)\right)$. This was performed for all channels, and a trial failing this criterion for any channel was then eliminated. The final time series was then concatenated in sequence.

1.5 ICA filtering

Independent Component Analysis of the data was performed using the standard EEGLab (Delorme & Makeig 2004) ICA analysis tools for each EEG data series, and in spite the DWT filtering, eye blink bioelectrical noise still could be seen in the ICA activations. A spatial localization test was made to detect eye blink related activations. If the ICA weight at the Fp1 and Fp2 channels were more than 75% of all weights, that activation was considered to be related to eye blinks and was removed from the data set. An activation was also flagged or removal based on two spectral tests: if its spectrum had large high frequency power, specifically, $P(20Hz) > P(10Hz)$, and if it had unrealistic power peaks with maximum power greater than the mean power plus three times the standard deviation. Once deleted, remaining activations were reassembled into channel EEG for further analysis. The same power criterion for trial elimination used directly on channel EEG data was also used on activation data produced by the ICA. This led to the elimination of a few trials that presented high variability. Like for the amplitude data, the ICA data was then concatenated.

2 Supplementary Figures and Tables

2.1 Supplementary Tables

Supplementary Table 1. Fourth order DWT nominal frequency bands.

Band	Fmin (Hz)	Fmax (Hz)	Source
1	50	100	High frequency electrical noise
2	25	50	Gamma brain waves
3	12.5	25	Alpha and Beta brain waves
4	6.25	12.5	Theta and Alpha brain waves
5	0	6.25	Delta brain waves and low frequency electrical noise

Note: Band 5 contains Delta waves and low frequency electrical oscillations from the power grid. Given the localized nature of wavelets, in this band is also most (but not all) of the energy of one-

time impulse signals from electrical or bioelectric origins like eye blinks. Bands 3 and 4 are composed of theta, alpha and beta waves. These two bands should contain most of the signal of interest. Band 2 contains most of the gamma wave power, and Band 1 should contain mostly electrical noise. Filtering was achieved by deleting Bands 1, 2 and 5 from the decomposed signal and remaining bands recomposed into a new time series. This procedure is exemplified on Fig. 2, showing a 10-sec excerpt of an EEG before and after the wavelet cleaning procedure.

2.2 Supplementary Figures

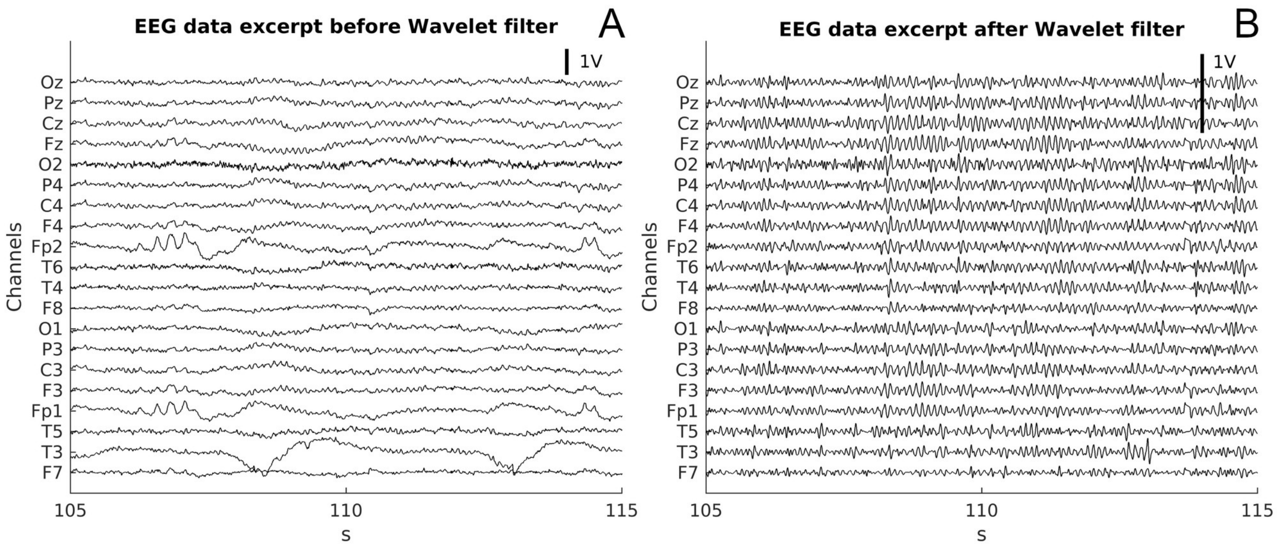

Supplementary Figure 1. Two 10-second EEG excerpts before (panel A) and after (panel B) Symlet9 filtering (Subject 2, Retest) are shown, voltage scale is shown by the vertical bar on the upper right corner. Only bands 3 and 4 were kept in the filtering (6.25 Hz to 25 Hz).

3 References

Al-Qazzaz N, Hamid Bin Mohd Ali S, Ahmad S, Islam M, Escudero J. Selection of mother wavelet functions for multi-channel EEG signal analysis during a working memory task. Sensors. 2015;15(11):29015–29035.

Calvo-Merino B, Gr`ezes J, Glaser DE, Passingham RE, Haggard P. Seeing or doing? Influence of visual and motor familiarity in action observation. Current biology. 2006;16(19):1905–1910.

Delorme A, Makeig S. EEGLAB: an open source toolbox for analysis of single-trial EEG dynamics including independent component analysis. Journal of neuroscience methods. 2004;134(1):9–21.

Faust O, Acharya UR, Adeli H, Adeli A. Wavelet-based EEG processing for computer-aided seizure detection and epilepsy diagnosis. Seizure. 2015;26:56–64.

Gandhi T, Panigrahi BK, Anand S. A comparative study of wavelet families for EEG signal classification. Neurocomputing. 2011;74(17):3051–3057.

Glassman EL. A wavelet-like filter based on neuron action potentials for analysis of human scalp electroencephalographs. IEEE Transactions on Biomedical Engineering. 2005;52(11):1851–1862.

Herrmann CS, Grigutsch M, Busch NA. 11 EEG oscillations and wavelet analysis. Event-related potentials: A methods handbook. 2005; p. 229.

Jones TA. Motor compensation and its effects on neural reorganization after stroke. Nature Reviews Neuroscience. 2017;18(5):267–280.

Malaver WJL. Wavelet multiresolution analysis and dyadic scalogram for detection of epileptiform paroxysms in electroencephalographic signals. Research on Biomedical Engineering. 2017;33(3):195–201.

Mamun M, Al-Kadi M, Marufuzzaman M. Effectiveness of wavelet denoising on electroencephalogram signals. Journal of applied research and technology. 2013;11(1):156–160.

Scolaro GR, de Azevedo FM, Boos CF, Waltz R, Andrade A, Pereira A, et al. Wavelet filter to attenuate the background activity and high frequencies in EEG signals applied in the automatic identification of epileptiform events. In: Practical applications in biomedical engineering. InTech; 2013. p. 81–102.

Subasi A. EEG signal classification using wavelet feature extraction and a mixture of expert model. Expert Systems with Applications. 2007;32(4):1084–1093.
