# Supplementary material for: Increasing Mu wave desynchronization after dance classes on people with Parkinson’s disease
Source: Front Hum Neurosci. 2025 Mar 24;19:1443730. doi: 10.3389/fnhum.2025.1443730 (PMC11973361; doi:10.3389/fnhum.2025.1443730)
Supplement: Supplementary file 2 [file Data_Sheet_2.pdf]

Baila Parkinson - Dance Session Planning Script

Theme: BAILAWEEN – the Baila Parkinson Halloween

ACT	WORKLINE	OBJECTIVE	MUSIC	DESCRIPTION
STRETCHING THE SKELETONS	Motor/Somatosensory	Warm up, stretching and voice exercises	Samhain - Mystical Celtic Music- Enchanting Witchcraft Music - Magical, Fantasy, Witchy Music	When entering the room, the participants will meet the teacher who will lead them through a warm-up and stretching exercise, standing up, slowly walking and impersonating choreographed characters in the style of horror stories (e.g.: the black cat, the skull, zombie, pumpkin, witch, vampire, black cat, monsters) in the sound of spooky songs and with vocal exercises (roars, screams, laments, ghostly sounds).
THE WITCH'S CAULDRON	Psychoemotional	Emotional expression/ Creativity/ Imagination / Voice exercises	Horror Themes Medley on Piano<br><br>Best Spooky Halloween Playlist - Haunted Graveyard Ambience Relaxing Halloween Music For Sleep	Introduction to the story, beginning of the Halloween walk: a story about a group of friends going to the forest for a seasonal festival, dancing by the fire (use stretching movements of great muscle groups and increasing joint range) ... and along the way they meet a witch with her cauldron. This Witch could only be defeated if people took the bad feelings inside themselves and poured them into the cauldron, what kept her strong were the bad feelings inside each one... every time they poured, the portal opens to a new phase...<br>Note: Each time a participant pours out their feeling, they will have to say it out loud along with the movement of throwing it, creating with the group a movement representing the feeling.
TRICK OR TREAT	Cognitive	Creativity/Imagination/Mimicking	Knock Knock, Trick or Treat? | Halloween Song	At this point, several pieces of paper with "trick" or "treat" written on them will be passed around on a pumpkin-shaped bowl. Each participant will take a piece of paper and must create a dance mime representing what is written on their paper. Without using words, the participants must, based on the identification of the movements, group themselves with participants who have similar identification. The group must then organize a choreographic sequence of movements to create the "trick" choreography and the "treat" choreography. These will be incorporated into the next act.
THRILLER	Motor	Motor coordination/Balance/Strength/Gait/Motor fluence/Memory	Thriller -Michael Jackson	The teacher will guide the group through Michael Jackson's classic choreography of Thriller, with adaptions for execution and the inclusion of the two choreographic pieces created in the previous act.
BOOO!	Socialization	Creativity/Imagination/Mimicking/ Group interactions/Voice exercises	Time Warp - The Rocky Horror Picture Show	The group will be divided into 3 to 4 groups to act out a scary story of their choice, presenting to everyone. The act will finish with the choreography of "Time Warp" from The Rocky Horror Show.
BAILAWEEN The Fantasy	Cognitive/Socialization	Cool down activity/Social interaction/Emmotional expression	The Rocky Horror Picture Show soundtrack	We will perform a Costume Contest. The costumes can be previously arranged with the participants, produced during the class, or just incorporated characters for the participants act and display. The group will judge and compete. Categories: Most Scary/ Least Scary / Creativity/ Dance & Acting. At the end gather the group to evaluate and share positive and negative points.
